# Home Enteral Nutrition in Adults—Nationwide Multicenter Survey

**DOI:** 10.3390/nu12072087

**Published:** 2020-07-14

**Authors:** Marcin Folwarski, Stanisław Kłęk, Agata Zoubek-Wójcik, Waldemar Szafrański, Lidia Bartoszewska, Krzysztof Figuła, Marlena Jakubczyk, Anna Jurczuk, Zbigniew Kamocki, Karolina Kaźmierczak-Siedlecka, Tomasz Kowalczyk, Bogna Kwella, Przemysław Matras, Karolina Skonieczna-Żydecka, Joanna Sonsala-Wołczyk, Jacek Szopiński, Krystyna Urbanowicz, Anna Zmarzły

**Affiliations:** 1 Department of Clinical Nutrition and Dietetics, Medical University of Gdansk, 80-210 Gdansk, Poland; 2 General Surgery Unit with Intestinal Failure Center, Stanley Dudrick’s Memorial Hospital, 32-050 Skawina, Poland; klek@poczta.onet.pl; 3 Nutrimed Home Nutrition Center, 35-201 Rzeszow, Poland; agata.zoubek-wojcik@nutrimed.pl; 4 Home Enteral and Parenteral Nutrition Unit, General Surgery Department, Nicolaus Copernicus Hospital, 80-803 Gdansk, Poland; ws2005@wp.pl; 5 First Department General and Transplant Surgery and Clinical Nutrition Medical University of Lublin, Home Enteral and Parental Nutrition Unit SPSK4, 20-954 Lublin, Poland; bartoszewska@o2.pl (L.B.); dr.matras@gmail.com (P.M.); 6 Nutricare Clinical Nutrition Center, 31-559 Krakow, Poland; krzysztof.figula@nutricare.com.pl (K.F.); tomasz.kowalczyk@nutricare.com.pl (T.K.); 7 Department of Anaesthesiology and Intensive Care Collegium Medicum in Bydgoszcz, Nicolaus Copernicus University, 85-067 Torun, Poland; marljakz@wp.pl; 8 Outpatient Clinic of Nutritional Therapy Clinical Hospital, 15-001 Bialystok, Poland; annamsacharczuk@gmail.com; 9 2nd Department of General and Gastroenterological Surgery Medical University, 15-276 Bialystok, Poland; zkamocki@gmail.com; 10 Department of Surgical Oncology, Medical University of Gdansk, 80-210 Gdansk, Poland; karolina.kazmierczak-siedlecka@gumed.edu.pl; 11 Department of Clinical Nutrition, Provincial Specialist Hospital, 10-561 Olsztyn, Poland; bogna-kwella@wp.pl (B.K.); krystynau@op.pl (K.U.); 12 Department of Human Nutrition and Metabolomics, Pomeranian Medical University, 70-204 Szczecin, Poland; karzyd@pum.edu.pl; 13 Clinical Nutrition Unit, Gromkowski Citi Hospital, 51-149 Wroclaw, Poland; joasiasw1702@gmail.com (J.S.-W.); aniazmarzly@gmail.com (A.Z.); 14 Department of General Hepatobiliary and Transplant Surgery, Collegium Medicum, Nicolaus Copernicus University in Torun, 85-094 Bydgoszcz, Poland; jacek.szopinski@wp.pl

**Keywords:** home enteral nutrition, tube feeding, artificial nutrition, home care

## Abstract

Home enteral nutrition (HEN) is an important part of the health care system, with a growing population of patients around the world. The aim of our study was to analyze one of the largest cohorts of HEN patients to provide the most recent data available in European literature. A multicenter, nation-wide survey in the period of 1 January 2018–1 January 2019 was performed in Poland. Data concerning adult patients on HEN in 2018 during 1 year of observation time were analyzed: demographic characteristics, primary disease, technique of enteral feeding, diet formulation and amount of energy provided. A total of 4586 HEN patients (F: 46.7%, M: 53.3%) were included in the study. The primary diseases were: 54.5% neurological (17.4%—neurovascular, 13.7%—neurodegenerative), 33.9% cancer (20.2%—head and neck, 11.7%—gastrointestinal cancer), 2.5%—gastroenterology, 1.5%—inherited diseases. Of new registrations in 2018—cancer patients 46.3%, neurological patients 45.1%. The median age overall was: 64 yr., BMI-20.2 kg/m^2^, NRS 2002 score—4.28. A total of 65% of patients were treated with PEG, 11.6% with surgical gastrostomy, 14.3% with naso-gastric tube and 7% with jejunostomy. Boluses were the most common method of feeding (74.4%). Gravity flow was used in 17.6% and peristaltic pump was used in 8% patients. The median energy provision was 1278 kcal/day and 24 kcal/kg/day. The most commonly used diets were: isocaloric (28.1%), protein-enriched isocaloric (20%) and protein-enriched hypercaloric (12%). The median overall duration of HEN was 354 days, 615 days for neurological and 209 days for cancer patients. A number of new registrations of cancer patients was significant and long duration of HEN in this group is encouraging. A developing spectrum of enteral formulas available enables more specified nutritional interventions.

## 1. Introduction

Home enteral nutrition (HEN) is a medical procedure provided to patients requiring nutritional support, unable to achieve nutritional goals with a standard oral, home-made diet, when the continuation of hospital stay is no longer necessary. It is considered to be a life-saving procedure. Primary diseases of HEN patients in most studies from Europe, US and Asia are neurological diseases and cancer; particularly of head and neck region. ESPEN (European Society for Clinical Nutrition and Metabolism) HEN guidelines published in 2019 described the indications for the home enteral nutrition [1]. The prevalence of HEN has been growing. Data from U.S. population showed an increase from 463 per million citizens in 1995 [2] to 248.846 adult patients and 1385 per million U.S. inhabitants latest study published in 2017 [3]. An Italian study showed an increase of HEN patients in 11-year epidemiological data and mean prevalence rate: 464 ± 129 cases/million inhabitants at home compared to 478 ± 164 in Nursing homes [4].

The cost-effectiveness of the procedure appears to be confirmed in specific groups of patients [5] though generalized and world-wide conclusions are harder to determine due to specific national characteristics. European survey showed that the reimbursement rate may be related to the economic status of the countries [6]. This specific correlation, however, was not confirmed in the Asia-Pacific region [7]. Studies show the important role of home nutritional support as a part of a multidisciplinary approach in the oncological treatment [8,9]. New therapies result in improved survival of cancer patients who become a dominant group of newly qualified HEN patients. A growing population of HEN patients demands a broader perspective on epidemiological data. Only a few European countries gather information on HEN in organized national or regional registries. Since the introduction of Home Nutrition in Europe there have been only several studies concerning this population of patients from Spain, UK, Italy, French and one large European survey from ESPEN– HAN–group [10,11,12,13,14,15]. The latest European epidemiological data come from 2016 [16]. Data comparison may be inconclusive due to various organizational structures of health care systems with different reimbursement programs and diverse populations of patients. In our opinion, several major factors from European studies are contributing to the heterogeneity of the HEN populations; co-payment rate of nutritional support, enrolment of patients consuming blenderized food or oral nutritional support in HEN statistics.

First polish centers provided HEN in 2003 already, but the reimbursement of the procedure has been available only from 2007. Polish data proved that a structured program of HEN funded by National Health Fund (the only and public Polish insurance company), with the use of commercial enteral formulas and support of specialized Nutrition support teams (NST), has improved clinical outcomes, decreased health care costs resulting in weight gain in patients, reduced incidence of infectious complications and the number of hospital admissions [5].

Considering polish qualification rules for reimbursement of HEN (only tube-fed patients with commercial enteral formulas, no funding for oral nutritional support) our population of patients allows epidemiological analysis in a relatively homogeneous population. Our study aimed to provide the most recent data concerning a cohort of HEN patients.

## 2. Materials and Methods

We conducted a multicenter nation-wide survey in 22 polish centers of HEN. The study was coordinated by the Section of Home Artificial Nutrition of Polish Society for Parenteral, Enteral Nutrition and Metabolism (POLSPEN). A questionnaire developed and approved by the study group was distributed among the centers. The study protocol was approved by Local Ethics Committee (KB-7/20). Adult HEN patients treated between January 1 and December 31, 2018 were included in the study. Patients on a reimbursed program of HEN by the National Health Fund under the supervision of nutritional support teams were enrolled. All patients were treated with commercial enteral formulas delivered via tube (no patients with oral nutritional support). Patients were organized into main groups according to the primary diseases: cancer, neurological, gastroenterological, inherited diseases and others. Newly registered patients for HEN in the observation period were elected for separate analysis.

Analyzed parameters were primary disease and indications for HEN, demographic characteristics, nutritional status, access route, diet type, energy intake, length of nutrition, reasons for termination of HEN. Diets were grouped according to the following definitions: hypercaloric ≥1.3 kcal/mL, protein enriched >4 g/100 mL, fiber enriched >5 g/L, standard polymeric and isocaloric. Access routes were percutaneous endoscopic gastrostomy (PEG), gastrostomy (surgical), jejunostomy, naso–gastric tube, naso–jejunal tube, percutaneous endoscopic gastrostomy/jejunostomy (PEG–PEJ).

The calculations were carried out with the use of Statistica 13 package and Microsoft Excel 2013. The descriptive statistics include averages, medians and standard deviations (SD).

## 3. Results

4586 patients were included in the analysis, 2620 received HEN in January 2018, and the next 1966 were enrolled to the procedure during observation time (1 year) (Table 1).

### 3.1. Primary Disease

Most of the patients in the overall population were qualified to HEN due to neurological diseases 54.5% and cancer 33.9%. During first point of prevalence in January 2018 main underlying diseases were neurology 61%, cancer 24.7% however, among new registrations—neurology 45.1% and cancer 46.3%. Dominating indication for HEN in neurological patients were neurovascular diseases (16.9% of all and 32.6% of neurological) and neurodegenerative (14% of all and 25.8% of neurological). Out of new cancer patients, most common were head and neck cancer (26.2% of all and 57% of cancer) and gastrointestinal (GI) cancer (17.2% of all and 37.3% of cancer) (Table 2). GI cancer patients were mostly; esophageal cancer (70% of GI cancer, 24% of all cancer and 8.26% of all patients) and gastric cancer (24% of GI cancer, 8.3% of cancer and 2.8% of all patients). From new qualifications: gastric cancer (29% of GI cancers) and esophageal cancer (65.4% of GI cancers).

Dysphagia was the most common cause for HEN (84%), 14%—mechanical obstruction of the GI tract, though in a subpopulation of cancer patients the obstruction of the GI tract was observed in 37% patients whereas dysphagia in 60%.

### 3.2. Technique of EN

**Figure 1:**
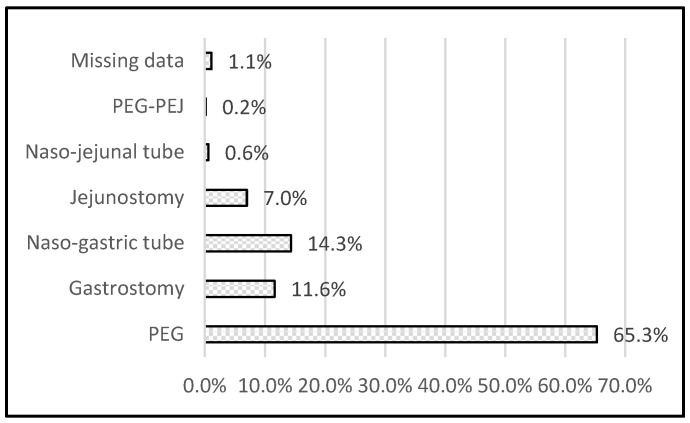
Access route for enteral nutrition. PEG—Percutaneous endoscopic gastrostomy, PEG–PEJ—Percutaneous endoscopic gastrostomy/jejunostomy.

The most common access route was PEG (65%) and naso–gastric tube (NGT) (14.3%). Of cancer patients, 13% were treated using jejunostomy (Figure 1). Bolus administration was most common (74%), continuous flow in 25.6% patients (pump or gravity) (Table 3).

### 3.3. Enteral Diets

40% of patients were treated with protein enriched enteral formulas, 17% with fiber rich, 16% were hypercaloric and 28% standard. Cancer patients received mostly protein enriched 43% diet, 24.1%—standard and 18% hypercaloric formula. Protein-enriched 38% and standard 30.8% diets were prescribed most often for neurological patients (Table 4). Mean amount of energy from enteral formulas administrated was 1278 kcal/day and 24 kcal/kg/day, cancer patients—1364 kcal/day, neurological patients—1223 kcal/day (Table 3).

### 3.4. Outcomes

The outcome after observation time is described in (Table 5). Most of the patients were still receiving HEN, 40.2% died during the observation period. Other reasons for HEN termination were resumed sufficient oral nutrition (5.3%), withdrawn consent (1.2%) or transfer to another unit. Median duration of nutrition in the overall population was 354 days, however, 48% of patients were still on HEN at the end of the observation time. The length of HEN in the group of patients who ended the procedure during observation period was 218 days. Neurological patients were those with the longest median 615 days while cancer patients—209 days. Head and neck cancer—218 days, GI cancer—190 (gastric cancer—96, esophageal cancer—222 days) (Table 5).

## 4. Discussion

To our knowledge, this is one of the largest and most recent epidemiological studies concerning HEN patients in Europe. It worth noticing that the population of Polish HEN patients has been growing rapidly and has been evolving. A previous study reported 31% of cancer patients in 2008% and 14% in 2013 [15]. Our analysis showed an increase in cancer patients to 33.9%. It could be noticed that among new registrations for HEN, cancer patients have become more prevalent. Cancer was the primary disease in 46% of patients qualified for HEN in 2018. A growing trend is particularly noticeable in the head and neck cancer population—4.5% cases in previous studies from 2013 [15] and 20% (26% of all new qualifications and 57% of new cancer patients) in our study. Likewise, GI cancer—5.2% in 2013% and 11.7% (17.2% of all new qualifications and 37% of new cancer patients). A similar tendency was observed in BANS report—new registrations with cancer receiving HETF has gradually increased from 25% in 2000% to 39% in 2010% and 43% in 2015, where head and neck cancer accounted for 77% of new HETF registrations with cancer in 2010 (67% in 2000)and 80% in 2015 [17,18]. Other European surveys reported neurodegenerative and neurovascular diseases to be primary diseases of 30%–67% patients, Head and neck cancer—7.5%–17.3%, GI cancer—7.1%–9.8% and cancers of other location—8.2%–15.3% [13,14,15,19,20].

Data reporting the mean duration of HEN is essential for designing health care policies and qualification rules, especially for palliative cancer care. ESPEN guidelines state that one month of expected survival should qualify a patient for the procedure [1]. Of all patients included in the study, 48.5% remained on HEN during observation time and 40.2% died. Median duration of HEN in our study was 354 days and only 7.9% of patients were treated less than a month. Even though the rate of cancer patients was significant in our study median duration of HEN has improved in comparison to previous reports. Klek et al. study showed a mean duration of HEN was 8.5 months in 2008 and 9.5 in 2013 [15]. Median HEN time of cancer (209 days) and neurological (615 days) patients was notably longer than in other previous publications. Cawsey observed the mean duration of HEN for: cancer patients—122 days, neurological disorders—187 and GI disorders—161 [19]. The median length of HEN according to Paccagnella was 196 days (neurovascular: 261 days, neurodegenerative: 251.5 days, head neck cancer: 118 days, abdominal cancer: 82.5 days, head injuries: 788 days, congenital pathologies: 387 days) [13].

A study by Wong concerning Asia countries from the Pacific region showed that countries lower-middle income use blenderized diets (40%) or both blenderized and commercial supplements (60%) [7]. Oley foundation reported 65.9% of HEN patients fed with blenderized food for an average of 56% of daily nutritional intake [21]. The Polish reimbursement program of HEN is designated for patients treated only with industrial enteral formulas using tube feeding. Therefore, patients on oral nutrition support nor with blenderized food were not included in the analysis. Previous studies showed that conversion from blenderized food to industrial formulas and HEN program with nutrition team support resulted in improved outcomes [5,22]. Most studies concerning HEN underline that long-term nutrition most often requires standard enteral formulas; however, certain medical conditions may involve protein, fiber or calorie enrichment as well as carbohydrate reduction for diabetic patients. We observed that only 24% of cancer patients were treated with standard polymeric diets, 43% with protein-enriched, 18% hypercaloric, 16% oligopeptide and 13% fiber-enriched formulas. Of the neurological patients, 30.8% consumed standard diets, 38% protein-enriched, 19% fiber-enriched and 14% hypercaloric. Previous Polish data from 2008 showed 96% of HEN patients with standard diets prescribed. In 2013 51% of patients were on standard diet, 12.9% on protein-enriched and 6.25%—energy-dense. This evolution may be explained by the increased rate of cancer patients requiring nutritional support during the post and preoperative period or during oncological treatment. Growing awareness of malnutrition and knowledge of clinical nutrition may be another explanation of the broader usage of specialized commercial diets. Spanish HEN registry showed a significant amount of patients on immunomodulatory formula (49.7%), normocaloric and polymeric formula administered in 24.9% of patients, a hypercaloric only in 5.4% of patients, protein-enriched and polymeric formula in 15.6% of patients, a semi-elementary formula in 1.6% of patients and fiber-enriched in 2.2%. However, it is worth mentioning that HEN was administered orally in 68.3% of participants [10]. In Spanish home care units patients in 18.3% of men and 16.3% of women received standard formulas and 19.6 and 13.7—specific diets [23].

The most common GI access for EN in our study was gastrostomy—77% (PEG or surgical gastrostomy) and bolus administration (74%). Similar results come from the latest BANS report (80%—gastrostomy) [17]. However, ESPEN HAN group reported 29.3% of patients with naso–gastric tubes [12] and Italian group with even higher prevalence of NGT (nearly 60% of neurological patients, 36% of Head and neck cancer and 23% with abdominal cancer) [13]. In Spanish reports most popular was the oral route, than NGT and PEG only in 6.8% [10] to 10% [24]. Italian data from 2012 show that epidemiological studies should be compared with caution since almost 55.4% of the patients included in the HEN summary were treated with ONS (reported as HEN in Italy till 2008). Moreover, there was a group of individuals receiving both HEN and HPN [25].

According to ESPEN guidelines, recommended energy provision ranges between 25–30 kcal/kg/day in a healthy population and similarly in cancer patients [9]. Median calorie intake in our study was close to recommended estimations (24 kcal/kg/day) similarly to cancer patients (24.2 kcal/kg/day). Comparable results were observed in the Italian study (24.4 kcal/kg/day) [13].

In the Cafane cohort, mean amounts of the enteral diet were 1644 mL (men) and 1686 mL (woman), respectively with no data concerning the calorie density of the diets [11]. UK survey on bolus tube feeding also confirmed the mean energy intake of 6272 kJ/day (ca. 1498 kcal/day) and 25 kcal/kg/day [16] in the HEN population.

### Study Limitations

There is no centralized registry for Polish patients on HEN therefore authors of the manuscript had to rely on voluntary work of the regional HEN units regarding completing the survey. The authors are aware of some limitations of the study. Centers reported primary diseases but provided no detailed information on the disease or cancer stage. Additionally, no data about enrolled patients who were transferred to another unit or continued enteral nutrition in the stationary palliative care unit was included. Authors hope, however, that the abovementioned limitations have not influenced the outcome of the study.

## 5. Conclusions

The study demonstrated a growing number of patients requiring HEN and an evolving patient profile. Though neurological diseases are dominant primary indications for HEN, a significant amount of new cancer patients is noticeable. The duration of HEN has also increased, which provide new and interesting insight into palliative care. A broad spectrum of commercial enteral formulas available for HEN enables personalized nutritional therapy and reaching the target goal for energy and protein intake. Repeated epidemiological studies based on structured registries are needed in the field of home nutrition.

## Figures and Tables

**Table 1:** Basic demographic statistics.

	Overall	January 2018	New Patients
N	*n* = 4586,100%	*n* = 2620, 57.1%	*n* = 1966, 42.9%
Age (mean ± SD)	64 ± 19	62 ± 21	67 ± 16
Male	53.30%	51.70%	55.60%
Female	46.70%	48.30%	44.40%
BMI (kg m^−2^)	20.2 (SD-4.3)	20 (SD-4.3)	20.6 (SD-4.3)
NRS 2002	4.28 (SD-1.17)	4.23 (SD-1.26)	4.36 (SD-1)

**Table 2:** Primary diagnosis.

	Overall	January 2018	New Patients
**Neurology**	54.5%	61.6%	45.1%
Neurovascular	17.4%	17.7%	16.9%
Neurodegenerative	13.7%	13.4%	14.0%
Cerebral palsy	4.3%	6.1%	1.8%
Multiple sclerosis	3.0%	4.2%	1.5%
Amyotrophic Lateral Sclerosis	7.5%	8.8%	5.7%
Other Encephalopathy	3.4%	5.2%	1.2%
Other neurological	3.8%	4.4%	3.0%
**Cancer**	33.9%	24.7%	46.3%
Head and neck	20.2%	15.7%	26.2%
GI	11.7%	7.6%	17.2%
Other (cancer)	1.8%	1.2%	2.6%
**Gastroenterology** ^1^	2.5%	2.6%	2.3%
**Inherited diseases** ^2^	1.5%	2.3%	0.5%
**Other** ^3^	7.5%	8.8%	5.8%

^1^ Gastroenterology: non-cancer gastric and esophageal strictures, motoric dysfunctions of upper gastrointestinal (GI) tract, Crohn’s disease, malabsorption, chronic pancreatitis. ^2^ inherited: neurofibromatosis, Down’s syndrome, muscle dystrophy, other rare inherited diseases. ^3^ other: mental disease, trauma, cardiac and respiratory insufficiencies, cystic fibrosis, malnutrition.

**Table 3:** Administration technique and energy provision.

**Administration Technique**	**%**
Pump	8.0
Bolus	74.4
Gravity flow	17.6
**Calorie provision**	**kcal/kg/day (median)**
All patients	24
Neurology	23.1
Cancer	24.2
Inherited diseases	27.8
Gastroenterology	24.4
Other	25.0

**Table 4:** Types of enteral diets.

Diet	All (%)	Cancer (%)	Neurology (%)	Other (%)
Standard	28.1	24.1	30.8	26.8
Protein-enriched	20.0	21.3	20.2	14.7
Oligopeptide, protein enriched	0.0	0.1	0.0	0.0
Oligopeptide, hypercaloric, protein enriched	0.1	0.2	0.1	0.0
Oligopeptide	10.0	15.4	6.9	8.7
Hypercaloric, protein enriched	12.0	13.9	10.4	14.2
Hypercaloric	0.8	1.3	0.5	0.8
Fiber-enriched, protein enriched, isocaloric	5.0	4.9	4.9	5.7
Fiber-enriched, protein enriched, hypercaloric	0.4	0.4	0.4	0.4
Fiber-enriched, hypercaloric	0.2	0.1	0.0	0.8
Fiber-enriched	11.6	8.1	13.4	13.0
Diabetic	7.8	6.8	8.5	7.0
Diabetic, hypercaloric, protein enriched	2.2	2.2	2.0	2.8
Diabetic, hypercaloric	0.3	0.2	0.2	0.9
Missing data	1.7	1.1	1.6	4.2

**Table 5:** Outcomes and median duration of home enteral nutrition (HEN).

Outcome (%)	All	Neurology	Cancer	Inherited	Gastroenterology	Other
Still on HEN	48.5	52.9	37.3	60.3	60.2	60.8
Death	40.2	37.3	49.2	29.4	23.0	28.3
Resumed sufficient oral nutrition	5.3	4.3	7.0	5.9	5.3	4.7
Resignation from HEN	1.2	1.1	0.9	1.5	2.7	2.9
Transfer to another unit of HEN	0.6	0.7	0.3	1.5	1.8	0.6
Transfer to stationary palliative care unit	4.1	3.6	5.2	1.5	7.1	2.7
Duration of HEN (median; interquartile range)	354; 1108	615; 1275	209; 534	1020; 1517	419; 1671	943; 1845
